# Implementing nutrition policy, system and environmental change strategies in a rural food pantry to improve nutrition security: contextual factors and outcomes

**DOI:** 10.1017/S1368980025101754

**Published:** 2025-12-26

**Authors:** April K. Hermstad, Lauren M. Bigger, Karen Ebey-Tessendorf, Gloria C. Simmons, Michelle C. Kegler

**Affiliations:** 1 Rollins School of Public Health, Department of Behavioral, Social, and Health Education Sciences, Emory University, Atlanta, GA, USA; 2 Georgia Department of Public Health, North Central Health District, Macon, GA, USA; 3 Hancock Helping Hands, Sparta, GA, USA

**Keywords:** rural health, food pantry, food insecurity, nutrition policy, policy systems and environmental changes

## Abstract

**Objective:**

To describe and evaluate nutrition-related policy, system and environmental (PSE) change strategies implemented in a rural, volunteer-run Georgia food pantry, exploring facilitators and barriers and changes in clients’ perceptions of food distributed following implementation of nutrition-related PSE changes.

**Design:**

The mixed-methods evaluation used pre-post key informant interviews, client surveys and programme documents to assess implementation and outcomes of a nutrition policy and other PSE changes.

**Setting:**

Hancock County, Georgia.

**Participants:**

Survey respondents were food pantry clients who completed surveys both in January 2021 and March 2022 (*n* 155). Key informants were programme staff, a local coalition member and food pantry leadership (*n* 9).

**Results:**

Nutrition-related PSE changes included a nutrition policy, produce procurement partnerships and enhanced refrigeration; an awareness campaign and nutrition education were also conducted. Facilitators included the implementation approach (e.g., encouraging small steps and joint policy development), relationship formation and partnerships. Barriers were modest capacity (e.g., funding and other resources), staffing/volunteers and limited experience with food policy and procurement processes. Client surveys in 2021–2022 showed canned/dried foods as most commonly received, with significant (*p* < 0.05) increases at follow-up in always receiving meat/poultry/seafood and significant decreases in always receiving canned fruits and dry beans/lentils. In both 2021 and 2022, substantial proportions of respondents reported food insecurity (>60 %), having obesity (>40 %), poor/fair health (>30 %) and a household member with hypertension/high blood pressure (>70 %).

**Conclusions:**

Nutrition-related PSE changes in rural food pantries to improve the healthfulness of foods distributed require substantial resources, yet if sustained, may increase client access to healthy foods and improve diets.

Food insecurity, defined as insufficient or inconsistent access to safe and nutritious food due to limited resources, impacts 37·2 million people (11·1 %) in the United States^(1)^. Rural communities experience higher rates of food insecurity than urban communities (14·7 % and 12·5 %, respectively)^(2)^. Food insecurity is linked to chronic disease and poor mental health^(3–6)^. These factors worsened during the COVID-19 pandemic among food pantry clients, with increased economic hardships and food needs resulting in greater psychological stressors^(7)^.

Many food-insecure households utilise food pantries to meet their food needs, but pantries experience challenges with meeting demand and providing nutritious foods^(8)^. Initially established as emergency food sources for families, food pantries have become primary, routine food sources for many. In 2020, 6·7 % of U.S. households reported using food pantries^(9)^. High prevalence of food insecurity in communities, increased demand, low access to healthy food and broader economic conditions have exacerbated pressures on food pantries^(10,11)^. A qualitative study of operational barriers in rural Mississippi identified growing demand for services, stricter reporting requirements and infrastructure (including limited physical spaces) as key challenges^(11)^. Additionally, food pantries have limited resources to both provide enough food to meet demand and ensure high nutritional quality^(12–14)^. While food pantries address food insecurity, the food they provide does not always meet nutritional recommendations,^(15)^ and clients’ reported consumption of fruits and vegetables is nutritionally inadequate^(16)^. Given the low-cost and relatively high availability of energy-dense, nutritionally poor food, food pantries struggle to address food insecurity without contributing to diet-related diseases^(17)^. Despite the availability of evidence-based approaches to improve the nutritional quality of foods provided in charitable food settings, pantries are often hesitant to restrict donations and typically rely on volunteers and have modest capacity to implement appropriate nutrition policies^(13,14,18)^. The increased use of food pantries during the COVID-19 pandemic further strained pantries through supply disruptions and reduced volunteerism^(19)^.

Despite challenges, nutritional policy interventions and other food pantry-based interventions including nutrition education, providing recipes and increasing fresh food have succeeded in addressing food insecurity, improving overall nutrition intake, educating on healthy food choice, supporting diabetes management and improving access to community resources^(16,20–22)^. A 2023 qualitative study identified five themes central to food pantries’ ability to implement healthy eating initiatives, including food pantry capacity and logistics, networks and relationships, community nutrition practitioner capacity, pantry user characteristics and stigma and stereotypes, highlighting the need for food pantry nutritional projects to consider both pantry- and community-level factors impacting implementation^(23)^. A 2015 systematic review of nutrition policy and environmental strategies implemented in rural communities noted three common approaches to strategy adaptation, including accommodating long distances to food sources, tailoring strategies to local cultural and food preferences and building strong local partnerships^(24)^. While this review is relevant, there is limited documentation specifically on how rural food pantries implement nutritional policy interventions.

Our study’s main objective was to describe the implementation and mixed-method evaluation of nutrition-related policy, system and environmental (PSE) change strategies in a rural food pantry in a predominantly African American Georgia county. Specifically, we describe the implementation of nutrition-focused PSE strategies, factors that facilitated or hindered implementation and clients’ perceived changes in foods distributed.

## Method

### Programme description and setting

**Figure 1.**
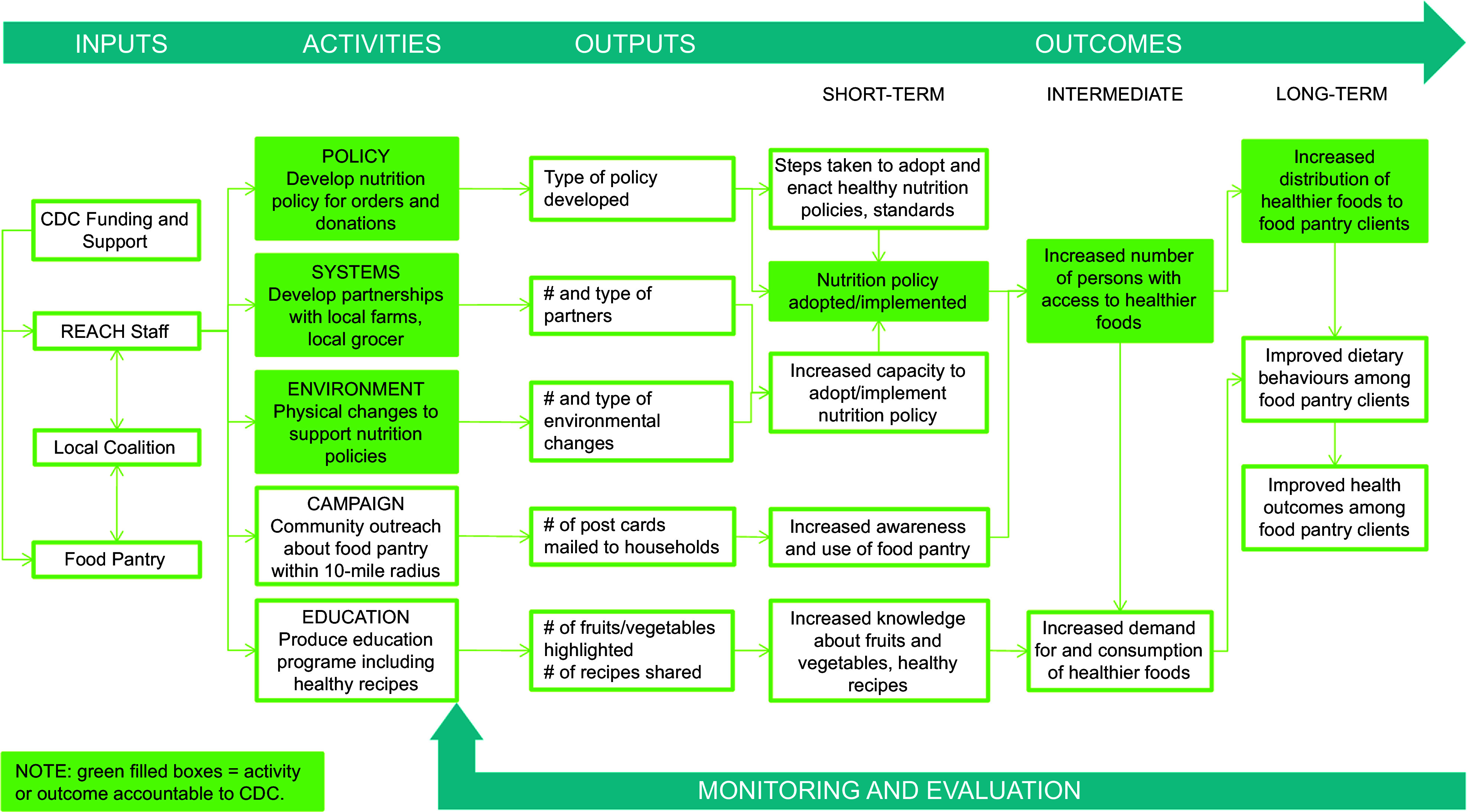
Logic model for implementing nutrition strategies in a rural food pantry.

From 2018 to 2023, Georgia’s North Central Health District was funded through the Centers for Disease Control and Prevention (CDC) Racial and Ethnic Approaches to Community Health (REACH) programme^(25)^ to implement culturally appropriate interventions among Black and African American residents of Hancock County, Georgia. Hancock County, located in rural north-central Georgia, has approximately 8500 residents, about 72 % identifying as African American or Black^(26)^. County Health Rankings^(27)^ data from 2018, the same year as the grant started, placed Hancock County 157th of 159 Georgia counties on economic indicators, 150th for health factors and 147th for health outcomes. Approximately one-third of Hancock County residents were living in poverty and 26 % were experiencing food insecurity^(27)^. The Hancock County REACH team, including Georgia’s North Central Health District staff and local health coalition members, implemented evidence-based strategies to address nutrition, increasing access to healthier foods through improving local food systems. A logic model (Figure 1), developed collaboratively with REACH partners and informed by CDC programme guidance, illustrates anticipated outcomes of the nutrition-related strategies. Activities include developing a nutrition policy, establishing systems for expanding healthy food sources (i.e., partnerships for produce procurement), environmental changes (i.e., enhanced refrigeration) to support policy implementation, an awareness campaign and nutrition education. Short-, intermediate- and long-term outcomes included policy adoption and implementation, increased access to healthier foods and increased distribution of healthier foods.

A key site for the Hancock REACH team’s nutrition work was a food pantry located 1-mile from the centre of the county seat. In June 2020, a formal agreement with REACH to work towards healthier food distribution commenced the collaboration with the food pantry. The food pantry received more than $30 000 in combined funding and related purchases to support their work with REACH over 3 years. The volunteer-managed food pantry is an important food source for county residents. From 2019 to 2023, the food pantry experienced substantial growth in the number of households served, with 408 households served (822 individuals, 9·4 % of county residents) in fiscal year 2020 and 626 households served (1250 individuals, 14·4 % of county residents) in fiscal year 2023, more than a 50 % increase in 3 years. Using a box model, the pantry prepares a set number of identical food boxes monthly, which are intended as a supplemental food source and not meant to provide households with food for the entire month. Clients pick up one box per month per household, on one of three distribution days.

### Evaluation design

This mixed-methods evaluation of Hancock REACH efforts focused on a nutrition policy and implementation of supporting PSE changes in a local food pantry. Process and outcome evaluation questions are tied to logic model outcomes (Figure 1), including policy adoption/implementation steps (short-term), priority population reach (intermediate) and extent to which efforts to establish PSE changes led to increased distribution of healthier foods to clients (long-term). This study focuses on PSE change implementation procedures, partners, facilitators, barriers, lessons learned and client perceptions of changes in food distributed.

### Data sources and measures

#### Programme documents

Programme documents included meeting notes, food pantry client records, nutrition policy documentation, memoranda of understanding, and grantee reports. Document review was secondary to key informant interviews and client surveys described below.

#### Key informant interviews

Semi-structured key informant interviews were conducted in late 2020 (*n* 5) before implementation of the food pantry’s nutrition policy and again in late 2022 (*n* 5) to capture details about PSE implementation and impacts. Interviews included five Georgia North Central Health District staff, one coalition member/food pantry volunteer and three food pantry leaders. One participant, a Georgia North Central Health District staff person, was interviewed at both time-points, totalling nine individuals interviewed. Interviews gathered implementer perspectives about planning and decision-making processes for strategy selection and partner development, perspectives on PSE approaches and other strategies, implementation challenges, facilitators, other contextual factors and key outcomes. Examples of questions asked include ‘The policy was officially implemented in March, 2021. What changed as a result of the guidelines? Did you change what went in the food boxes? Were there any negative consequences or downsides to implementing the guidelines?’ and ‘Do you think you will be able to continue this work once the REACH funding ends?’ Interviews were conducted via Zoom or telephone, and audio recordings were used to produce transcripts for analysis.

#### Food pantry client surveys

A baseline food pantry client survey was administered in January 2021 (*n* 292, 58·4 % response rate), before nutrition policy implementation in March 2021. A 1-year follow-up survey was conducted in March 2022 (*n* 287, 62·5 % response rate). The present study includes only respondents who completed client surveys at both time-points (*n* 155). At both time-points, a packet containing a promotional flyer, survey and pre-addressed/pre-paid return envelope was placed in each food box distributed throughout the month. The survey cover page explained the purpose and procedures, including eligibility (one adult per household), confidentiality and compensation. Clients who returned their completed survey received a $20 gift certificate to the county’s only grocery store. Key survey measures are described below. Whenever possible, the survey was composed of validated and/or widely used measures.

#### Food received at the food pantry

To assess the frequency of fourteen foods across five categories received in the food boxes at baseline and follow-up, we asked ‘How often did you receive each of the following foods from [food pantry] during the past 6 months?’ Food items were adapted from Long et al.^(15)^ and Healthy Eating Research Nutrition Guidelines for Charitable Food Systems^(28)^. The foods, by category, were (1) fruits and vegetables, (2) grains, (3) proteins, (4) dairy and (5) other foods. Response options were always/almost always, sometimes and rarely/never. Table 5 provides a comprehensive list of foods in each category.

#### Food selection preferences at the food pantry

Using the same set of foods described above, respondents indicated whether they wanted more, less or no change to the selection of foods available at the food pantry (‘If you could change the selection of foods available at the pantry, what would you change?’).

#### Food insecurity

A two-item screener^(29)^ measured past-year individual-level food insecurity: ‘I worried whether my food would run out before I got money to buy more’ and ‘The food I bought just didn’t last and I didn’t have money to get more.’ Response options were never, sometimes or often. Those responding sometimes or often to at least one statement were classified as food insecure.

#### BMI

Self-reported height and weight^(30)^ were used to calculate BMI following CDC’s formula for calculating adult BMI [weight (lb)/[height (in)]^2^ × 703]^(31)^.

#### Chronic conditions in the household

To assess household chronic disease status, we asked: Has a doctor ever told anyone in your household that they (or you): (a) Have type 2 diabetes or high blood sugar? (b) Have hypertension or high blood pressure? Response options were yes or no^(32)^.

#### Health status

Respondents rated their general health status as excellent, very good, good, fair or poor^(33)^.

#### Demographics

Measures were adapted from the American Community Survey^(34)^ and Behavioral Risk Factor Surveillance System^(30)^ and re-categorised as needed. Demographic characteristics included age in years (18–24, 25–34, 35–44, 45–54, 55–64, 65–74 and 75+), gender (female, male), race/ethnicity (African American/Black not of Hispanic origin, White not of Hispanic origin and other), educational attainment (8^th^ grade or less, some high school, high school or GED certificate, some college or technical school and college graduate/post-graduate/professional degree), employment status (working full-time, working part-time, retired, not employed/homemaker/student/on disability and other), annual household income (<$10 000, $10 000–$25 000, $25 001–$50 000, >$50 000, don’t know and prefer not to answer), household size (open-ended) and children living in the home (yes/no).

### Data analysis

#### Programme documents

We examined numerous programme documents to understand food pantry operations and implementation of PSE changes at the food pantry. Relevant information from programme documents was abstracted to fill in gaps not answered by other data sources.

#### Key informant interviews

We created a codebook to group interview text by major topics (relationship formation, PSE strategy descriptions, facilitators, barriers, lessons learned, other considerations and factors for sustainability). Transcripts were coded independently by two coders, and discrepancies were resolved through consensus. NVivo 12 was used for data management and analysis. Thematic analysis was performed on coded text to identify themes, and matrix-style tables helped identify patterns by participant role and year.

#### Food pantry client surveys

We conducted descriptive analyses including frequencies and cross-tabulations on demographic and key outcome variables. We used McNemar’s test to assess whether there were significant changes in key outcomes from pre- to post-implementation of the nutrition policy. A threshold of *P* < 0.05 was used to determine statistical significance. All analyses were conducted in SPSS 29.0.

## Results

The results are organised by key logic model activities (e.g., develop nutrition policy, partnerships for produce procurement and environment changes) described in Figure 1.

### Developing and implementing a nutrition policy

**Figure 2.**
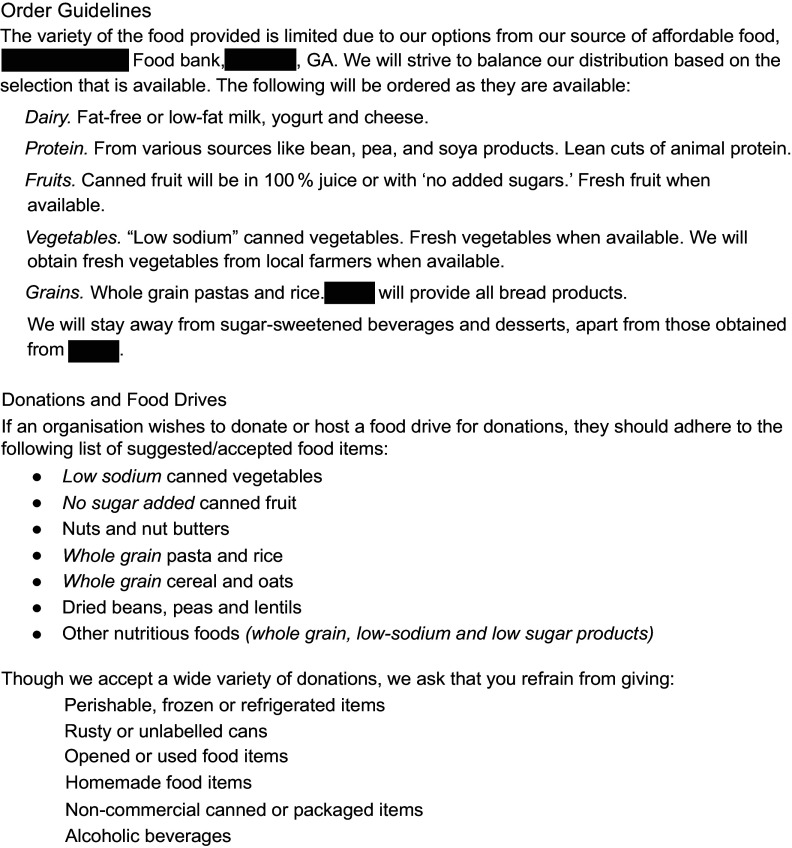
Food pantry nutrition policy.

Policy implementation, a short-term outcome, began in March 2021, including order and donation guidance based on USDA’s MyPlate guidelines for dairy products, fruits, vegetables, proteins and grains (Figure 2). Interview participants named several facilitators of policy development and implementation (Table 1). Several interviewees described the importance of relationship formation, beginning with REACH staff members regularly volunteering with the food pantry, preparing and distributing food boxes, observing and asking questions of pantry volunteers and leadership. This enabled them to learn about pantry operations, clientele needs and preferences and build relationships with pantry leaders. The partners were able to build a relationship based on mutual benefits of working together, including shared objectives and responsibility. The relationship was formalised with a signed memorandum of understanding, which concretised each partner’s responsibilities. REACH staff described their implementation approach including efforts to ensure food pantry partners were comfortable with the changes they committed to implement, encouraging small steps for change and building on existing efforts to ensure the work was feasible. After reviewing a range of existing policy examples, partners agreed on a policy that codified what the pantry was already doing and expanded on those efforts. The policy was jointly written and implemented with flexibility to avoid alienating major donors and reflected charitable food system limitations (i.e., availability of healthy foods at the food bank). Finally, strong partnerships facilitated the work. The food pantry, its leadership and volunteers had deep ties to the community they served. REACH staff had relevant professional and lived experiences that supported them in their roles. Finally, the partnerships formed an ever-increasing network of entities throughout the community creating broader momentum for change.

**Table 1.** Policy facilitators and barriers

Theme	Representative quotes
**Facilitators**	
Importance of REACH staff volunteering with the food pantry	They had to know how we were working, what we were doin’ with our organization. Then they had to look at, okay, this is what we plan on doin’. Can you all implement these things into y’all’s program? We worked together with lookin’ at what they could do, how they could help us. (FP1) I came in strictly as a volunteer. If you need me to carry boxes, if you need me to do heavy work, if you need me to be on the line packing boxes, whatever you need me to do, and then once that rapport is established, now I can kind of say well, I kind of wanted to do this today. (R4)
Highlight mutual benefits of working together	There were lots of discussions around healthy nutrition standards and making it make sense for us as the program workers, so that it could make sense to the community. (R2) I personally see a win-win opportunity for everyone with regards to the partnership with REACH being involved with [the food pantry] and REACH working together because no one entity can meet the needs of a local community anywhere. What [the food pantry] was doing when REACH became involved with them gave the folk who oversee REACH an avenue through which they could meet their objective, which helps [the food pantry] reach its objectives which ultimately helped the citizens to be able to have healthier meals that they’re able to serve. It’s a win-win all around. (FP2)
Formalise relationship with MOU	So [the MOU] puts responsibilities both for the REACH program and for the [food pantry], so it clarifies kind of the purpose and the expectations, roles and responsibilities. (R1)
REACH staff encouraged small steps for change/ built on existing efforts	They have been doing some things towards [nutrition policy], but they really haven’t classified it as such. So it’s good for them to know hey, you’ve already been doing something, so you’re ahead of the game and recognizing and appreciating the progress that they have already made. So that keeps them interested in [nutrition policy], knowing that you’re not really at the starting gate, you’ve done some things, let’s see how we can turn that into the real thing. (R2)
Joint policy development	He [FP partner] just took the initiative to do it. He was like, oh, it was only a couple of sentences. I was like but those were the sentences we needed. I said I literally added some formatting and a few sentences, but you gave me everything I needed. So, it wasn’t an issue for them. (R4) We had meetings about it. Actually, one Saturday I came up and wrote the nutrition policy… She got right on it on a Saturday and corrected things and added some things to it. It worked good. (FP2)
Policy written with flexibility	I think the policy was written with enough exceptions, so to speak, that I think they’ve been able to implement the policy. They did allow for the continuing donations from [grocery chain], which are bread items and also sweet dessert items. That is really the only sweet items that go in the food boxes, are those desserts from [grocery chain]. Because they wrote the policy, I think, in such a way that they felt certain they’d be able to implement it, they have been able to. (R1)
Community ties of food pantry and leadership/ volunteers	When [the food pantry] was established over 25 years ago, I was workin’ in public health…When they started that program, they had a board. They compiled a board. I was one of the individuals that was on the board at that time. I’ve known about it since its infancy. (FP1) We have some very dedicated and committed volunteers. We can depend on them because there’s a lot of work to be done to get food there and in those boxes and in their hands. We couldn’t do it without the commitment of the volunteers who come and work with us. (FP2)
REACH staff team with relevant professional and lived experience	I taught classes in the community, trying to target the low-income families that needed to know about nutrition, needed to know that there are simple recipes out there to feed their family. (R5) What worked in our favor is that before taking on this role, I volunteered at [the food pantry]. I had a chance to – for that firsthand experience where I really wasn’t looking to see how – I was just a part of the experience. So looking at some of the local considerations were how the pantry is currently operating, what the perception of the community is as far as the services of the food pantry. (R2)
Broader momentum for change in community	The people who we’re working with has the power of the coalition, so we’re not just working with [the food pantry] on [nutrition policy], we’re also working with the city for Complete Streets and to make it a more attractive area. (R2) So it’s happening in different places in the community. We even had a community member to offer up his private land for a community garden. So people are starting to think community garden, healthy, health sustaining. And we believe that we’ve influenced that to some degree. (R2) There are some organizations that want to work on nutrition as well, and they are always looking to partner, so that’s good. (R4)
**Barriers**	
Community change takes time	We may not be the healthiest county in Georgia by the time this program hits its five years, but I expect we will have made progress. (R2) So I mean, it’s a – it is kind of a continual changing – taking small steps and trying to make those small changes. (R1)
Lack of consistent funding to sustain the work	They worked with us to get that grant together. We implemented it. We put everything in it that we were supposed to put in it. Then we didn’t get it. That was disappointing. (FP1) Like we’ve dropped (dollar amount) in our checkin’ account in the last six months. Some of that was infrastructure, but it can’t go on. We went for years and years increasin’ our checkin’ account, but now it’s goin’ in the opposite direction… payin’ the local farmers has taken away a lot of resources that we had. I don’t say it’s a bad idea, but we’re gonna have to have some more funding. (FP2)
Limited resources/ underserved, large rural community	In rural communities, just be aware of your population that you’re serving and just know that what may work in a more populated and more accessible area may not work in a community like ours. We don’t have that many resources, so when we’re told that we have to do certain things in a specific way, it’s kind of a push back. (R3) The people are volunteers, they’re not paid, but they put in hours beyond just being there to give food to folks. So we really had to consider the volunteers, the work environment for the volunteers, the relationship with the food bank. (R2)

Interview participants shared barriers to policy scope and pace of development (Table 1). They considered the acceptability and appropriateness of the policy for their community, noting that change takes time, particularly to build trust and ensure that the larger community understood the vision. As a food pantry in a rural community, they faced a double barrier of limited resources in terms of funding and human capital/capacity. COVID-19 impacted the pace of policy development as REACH staff members were reassigned during the first year of the pandemic to address emergent public health needs, thus slowing progress.

### System changes to support nutrition policy implementation

System changes implemented at the food pantry (Table 2) focused on enhancing available produce options. The REACH team monitored nutrition policy implementation by reviewing monthly orders, tracking the nutritional content of regularly ordered items and making suggestions to improve availability of healthy food. The REACH team established relationships (*n* 4) with a local independent grocer, a regional produce wholesaler and two local farms to donate or sell healthy food options and locally grown produce to the food pantry. The REACH team provided funding support by covering costs of added produce in the food boxes. In the grant’s final year, the REACH team provided grant writing and fundraising support to the food pantry to help sustain the policy and related costs beyond the grant period. To be able to implement the policy, pantry leaders ordered healthier foods from the food bank when available. The partners communicated regularly with the regional food bank to understand food sources and existing nutrition standards. Finally, the food bank that supplied the food pantry with most of its food supported health promotion efforts, enabling the pantry to develop and implement a nutrition policy. The partners worked with the food bank to ensure implementation feasibility. Additionally, the food bank offered support by increasing healthy offerings and sending greater quantities of fresh produce to the pantry. Barriers to implementing these system changes included limited quantities of produce available at the food bank, especially early on, and limited capacity of small local farms to grow produce in the amounts needed to support the food pantry.

**Table 2.** Facilitators and barriers to system and environmental changes

Theme	Representative quotes
**System change facilitators**	
Relationships with food vendors to provide local produce	We partner with our local farmers. That’s one of the things that—a part of what REACH had done. They had coordinated lookin’ into the local farmers here that could be able to provided produce for us. That has been a positive thing for us too. It has been such a great help with the local farmers that were able to provide items for us. That made a difference. (FP1)
Funding support to pay additional produce costs	One of the things that I see coming from the partnership with REACH is some additional funds to help us better serve the public, particularly fresh fruits and vegetables. That support is phenomenal, I believe. (FP3)
Pantry staff ordered healthier foods	So I think it’s been a little bit sobering to realize that there’s a lot of parameters to making those choices and I do think that they are making as good choices probably as they can. (R1)
Food bank support	We were able to add grapes to the items, to the boxes. [The food bank] would get things like lettuces, strawberries, box of lettuces for a salad…Those are the kind of things that were extra. They said, well, we have these items. We’ll just send this along since you have refrigerator space to put those in. (FP1)
**System change barriers**	
Lack of FV at food bank	I do think there could potentially be months where they would not be able to have a fresh item. There are sometimes opportunities for frozen vegetables that come from the regional food bank. I think they’re always looking for whatever the options are. The cost of buying fresh produce or from farmers or from the local grocery store or from another place, the cost of that produce may be more than they spend on everything else. (R1)
Lack of growing capacity among local farmers	We really hadn’t been able to identify farmers that had that amount of produce available, you know, at the very specific times that they were needed. (R1)
Increasing client volume	We got enough money, though it’s goin’ down really quick. I think this last month, November, we did 600 people.…and this month it seemed like everybody came out of the woodwork and signed up… They’ve been increasin’, I don’t know, for six, seven months. (FP2)
**Environment change facilitators**	
Enhanced refrigeration space	I think probably the bigger thing was the fact that they had more storage. I think the policy and conversations we’ve had in about fresh fruits and vegetables has probably played a role as well. Previous, they simply didn’t have enough refrigeration space to do that on a regular basis. (R1)
Near elimination of sweets and salty snacks	I think probably the biggest thing that people would notice is there were no longer the salty snacks or the sugary snacks. They would occasionally have sugary things from the food bank as well, so those things were essentially eliminated. (R1)
**Environment change barriers**	
Rural nature limits food orders/deliveries	Because it is a rural food pantry, they get one truck load per month. They’re balancing volume, weight, what they can afford, and what the food pantry has available at that point. (R1)
**Campaigns/education facilitators**	
PSE change and education as complementary approaches	I think even us as health professionals, we have the knowledge but it doesn’t necessarily transfer to (laughs) good choices and good behavior. So I do think that policy changes and environmental changes can—can make a difference. I think making changes in the food pantry and in churches are one piece of it. There’s certainly other pieces in terms of schools or workplaces…I definitely think that people’s environments and the easy choices around them, and also the community norms and family norms play a big part. So it’s looking at how to change individual behavior by changing environments, by making things easier, more available, more affordable, more accessible, working to change community, organizational, and family norms. (R1) Given the community that we’re serving and the history of diets that African Americans may have, and because that’s the primary population that we’re serving, I think it’s better to start small than just going into a church or a business saying that, okay, we want you to change your policy and they need to just say that okay, you’re not eating healthy, we need you to change this in order for us to work with you. I think an education-based system may be better and then once they become more comfortable and more knowledgeable about the system changes, then they’ll be open to having that policy. I think that’s the better route. (R3)
Awareness campaign and other marketing efforts helped increase reach	We did a campaign for [the food pantry] in order to—for a ten mile radius, so people are aware of the pantry’s existence and that worked really well for us. (R2) I do think the profile of [the food pantry] has been raised in the last three years since we’ve been collaborating with them partly by helping them with putting articles in the newspaper, mentioning them on Facebook posts. Then the new signage that’s right there on the main highway, that is much more attractive and prominent than what was there before. I think the numbers of people coming have increased partly due to the marketing. (R1)
Adapted existing school-based produce programme for food pantry	So it’s something that is implemented in the school system here…and it was kind of a reiteration of what the kids see in school. They get to see it at school, they learn about it, they try it at school. Let’s bring it to their homes. So that’s how we started with doing Harvest of the Month for the food pantry recipients. (R4)
The food pantry is a key central location in the county	One benefit is to be able to meet with folks in a central location because we publish where we’re going to meet. Folks know that we’re gonna meet on those days. They make arrangements either with someone to pick them up and bring them or someone to pick up their food and take it back to them. That’s one of the things that happens in a rural community because it’s hard to get to folk. (FP3)
**Campaigns/education barriers**	
Challenging to reach people with information	Trying to build a communications network, trying to find out really how people get information, you know, where they go to for information. We live in a very large county, people’s habits are inconsistent. Some of the community events that we have counted on did not take place. (R2)
COVID-19	Before COVID we were doing food demonstrations also. (HHIP1)

### Environmental changes to support nutrition policy implementation

A few key environmental changes were implemented in the food pantry, supporting policy implementation and system changes (Table 2). A major environmental change was the installation of two industrial-size refrigerators, donated by the food bank. This enhanced storage increased the food pantry’s capacity to store perishables, including fresh produce and dairy. Beyond increasing healthier foods, another environmental change was the near elimination of sweets and salty snacks distributed, further supporting the policy. Interviewees described clients’ appreciation for the increase in fresh produce and other healthy foods, and clients promoted the changes to others through word of mouth. Interviewees hoped the changes would lead to improved client diets. The rural nature of the community presented major limitations for food orders and deliveries. In this case, the food pantry could place one order per month from the regional food bank located 65 miles away, balancing food cost, volume, weight and perishability.

### Awareness campaign and produce education

Interviewees discussed the importance of communicating about changes at the food pantry as a form of education. Multiple REACH staff described education as a complement to the structural changes and vice versa. One interviewee believed that structural changes without education is insufficient to obtain community buy-in or change client behaviour, and another espoused the alternate view that education alone is insufficient to change health behaviour without structural change. The REACH team and food pantry leaders implemented individual-level programmes, including an awareness campaign, other ongoing marketing efforts and a produce education programme (Table 2). The campaign, intended to increase awareness of the food pantry among residents (and required by the food bank), entailed a one-time postcard-mailing to all households within a 10-mile radius of the food pantry (*n* 3277) in Year 2, just before the COVID-19 pandemic. Other marketing efforts continued throughout the grant period including newspaper articles, social media posts and improved highway signage. Interview participants suggested that these efforts were effective in increasing reach to the community and pantry use by eligible households. The produce education programme, Harvest of the Month, was a simple adaptation of an existing school-based programme. It highlighted a different fruit/vegetable each month, which was provided in the food box with healthy recipes. COVID-19 ended the nutrition programme component involving in-person recipe demonstrations and taste samples.

### Lessons learned

Reflecting on their experience implementing nutrition-related PSE changes, interview participants discussed lessons learned (Table 3). General observations included needing to tailor PSE changes to a setting and community, understanding the charitable food system and working effectively with others to reach shared goals. Some participants discussed the need for outsiders to learn about the prioritised community before proposing solutions. Participants discussed the importance of mindset for external partners working in the food pantry space, including a desire to serve, demonstrating a positive attitude and treating others with respect and dignity. Additionally, some described the importance of supporting local farmers as partners and paying them accordingly. Finally, REACH staff and food pantry leaders expressed that some anticipated barriers did not arise, and the work was easier to implement than expected.

**Table 3.** Lessons learned

Theme	Representative quotes
Understand the charitable food system	Learning more about the bigger picture of the charitable food system, the access to the food, the cost of the food. (R1) Having the experience of working in a food pantry in my community, with people I know and seeing people, you know, that I know. So that gave me a great perspective… just reading, you know, how do food pantries work in tribal communities, how do they work in urban environments, how do they work in other rural communities like ours. Just absorbing everything I could about food pantries. (R2)
Collaborate and work effectively with others	I would encourage any organization in a rural community to work with other—if there are other food pantries, as an example, that exist there, then work with that food pantry to help strengthen what they’re doing by helping to use that as a way to get their information out. Then that information is being passed on to additional families, so we’re able to reach more people with more resources by working together. (FP3) We have been able to collaborate with other organizations, too. When people need funding for if their lights are being turned off or whatever, we refer ‘em to [a local agency]. There are people that—we know of other agencies that can try to help them. (FP1)
Get to know community where you want to work	Understanding the area that you work in, understanding the people that you’re working with, that’s important. You have to know your community and know what their needs are and try to meet their needs and not your own. (FP1) Spending time getting to know the people and the process and what they’re already doing before really proposing changes I think would be the main thing. Because that also shows—shows respect. Us outsiders don’t know everything (laughs). A lot of times know very little. (R1)
Treat all involved in this work with respect and dignity	Get involved with the local farmers and have the funding to pay them whatever they’re askin’ for. (FP2) It’s the way that you present yourself to people so that they will maintain their dignity as they come through because if you don’t allow a person to maintain their dignity, they gonna tell somebody else and they’re not gonna wanna come. I feel like we’ve been doin’ that because our numbers are increasin’ not decreasin’. (FP1)
Changes easier to implement than expected	It’s been a win-win situation. Well, we have to do all these reports now, and that’s per the USDA. I just thought it would be more reports, more involvement…We were able to use all of it that we did through [the] food bank through the USDA. I just copied it to the REACH Program. It was not a whole lot of extra work. (FP2)

### Perceived access to healthier foods

The key intermediate outcome was increasing the number of persons with access to healthier foods. In 2021, most survey respondents were ≥65 years (55·6 %) and nearly three-quarters were female (73·0 %; Table 4). Nearly all respondents identified as African American/Black (95·4 %) with 3·9 % identifying as White. Nearly half of respondents received a high school diploma or equivalent, and while 14·2 % reported some college or technical school, only 6·4 % reported completing college or receiving a post-graduate/professional degree. Most respondents were either retired (49·7 %) or not working, a homemaker, student or on disability (37·1 %). The vast majority of respondents reported incomes of ≤$25 000 (92·5 %). Households typically consisted of one (41·9 %) or two people (32·9 %), and one-fifth had children under age 18 living in the home (19·4 %). A large majority of respondents reported visiting the food pantry monthly (93·4 %) and had been using the food pantry for more than a year (75·2 %), preceding COVID-19. In the 2022 survey, we used more detailed categories for employment status, separating ‘on disability’ from ‘not employed/homemaker/student.’ This resulted in nearly one-third of respondents reporting disability status (32·9 %) and 5·2 % reporting that they were not employed, a homemaker or student. It is likely that proportions were similar in the 2021 sample.

**Table 4.** Client survey demographics (*n* 155)

	Baseline (2021)	
	*n*	%
Age		
18–34	6·	3·9
35–64	62·	40·5
65+	85·	55·6
Gender—female	108·	73·0
Race		
African American/Black	145·	95·4
White	6·	3·9
Other	1·	0·7
Education		
8^th^ grade or less	16·	10·4
Some high school	33·	21·4
High school or GED certificate	73·	47·4
Some college or technical school or above	32·	20·6
Employment		
Working full-time or part-time	20·	13·2
Retired	75·	49·7
Not employed/Homemaker/Student/On disability	56·	37·1
Income*		
<$10 000	65·	54·2
$10 000–$25 000	46·	38·3
$25 001–$50 000	8·	6·7
>$50 000	1·	0·8
Household size		
1 (self)	65·	41·9
2	51·	32·9
3 or more	33·	21·2
Children in the home—yes	30·	19·4
Frequency of food pantry use		
Once per month	141·	93·4
Once every few months	8·	5·3
1–2 times in last 12 months	2·	1·4
Length of food pantry use		
This is my first time	2·	1·3
<6 months	7·	4·7
6 months to 1 year	28·	18·8
1–2 years	25·	16·8
2–5 years	49·	32·9
>5 years	38·	25·5

* Percentages do not add to 100 % due to missing data (including responses of ‘don’t know’ and ‘prefer not to answer’).

Table 5 shows the percentages of respondents reporting always/almost always receiving fourteen types of foods in their food boxes at baseline and follow-up. In 2022, there were some statistically significant differences in reported frequency of always/almost always receiving certain foods versus 2021. The frequency of always/almost always receiving canned fruits low in sugar or syrup declined from 76·4 % to 54·0 % (*P* < 0·001). The percentage of respondents reporting always/almost always receiving dry beans or lentils declined from 80·7 % to 69·2 % (*P* < 0·01). Reports of always/almost always receiving meat, seafood or poultry (without breading and not fried) increased from 25·5 % to 44·0 % (*P* < 0·001). At both time-points, canned/dried foods were the most frequently reported food always/almost always received.

**Table 5.** Pre-post policy implementation changes, key survey outcomes, 2021–2022 (*n* 155)

	Baseline (2021)		Follow-Up (2022)		*P* value
	*n*	%	*n*	%	
**Frequency of foods ‘always or almost always’ received at the food pantry**					
a. Fresh fruits	50·	33·6	45·	30·4	NS
b. Fresh vegetables	45·	30·6	50·	34·7	NS
c. Canned vegetables low sodium (not including beans)	117·	78·5	108·	74·0	NS
d. Canned fruits, low sugar/light syrup	113·	76·4	81·	54·0	*P* < 0·001
e. Frozen fruits and/or vegetables	32·	22·2	44·	29·9	NS
f. Whole grains: bread, cereal, rice or pasta	109·	75·2	107·	73·8	NS
g. Nuts or seeds with nothing added or peanut butter	89·	59·3	85·	55·9	NS
h. Lower-/low-sodium canned beans	88·	59·9	95·	62·9	NS
i. Dry beans or lentils	121·	80·7	99·	69·2	*P* < 0·01
j. Meat/poultry/seafood without breading and not fried	37·	25·5	66·	44·0	*P* < 0·001
k. Low-fat, 1 %, or skim milk	25·	16·8	31·	21·2	NS
l. Low fat, 1 % or skim cheese	31·	21·7	24·	16·9	NS
m. Salty snacks: chips, pretzels, crackers, popcorn, etc.	17·	11·7	13·	9·0	NS
n. Desserts or sweets: ice cream, frozen yogurt, chocolate, cookies, cakes, snack cakes, donuts, pastries and candy	39·	25·7	41·	26·8	NS
**Health-related measures**					
Food insecure	95·	61·3	98·	63·2	NS
BMI*					
Underweight	3·	2·0	3·	2·0	NS
Healthy	33·	21·3	28·	19·0	NS
Overweight	46·	29·7	46·	31·3	NS
Obese	66·	42·6	69·	46·9	NS
Chronic conditions—Has a doctor ever told anyone in your household that they (or you):					
Have type 2 diabetes/high blood sugar	54·	34·8	55·	35·5	NS
Have hypertension or high blood pressure	113·	72·9	112·	72·3	NS
Poor/fair health status	48·	31·2	53·	35·3	NS

NS at *P* < 0·05.

* Percentages do not add to 100 % due to missing data.

**Figure 3.**
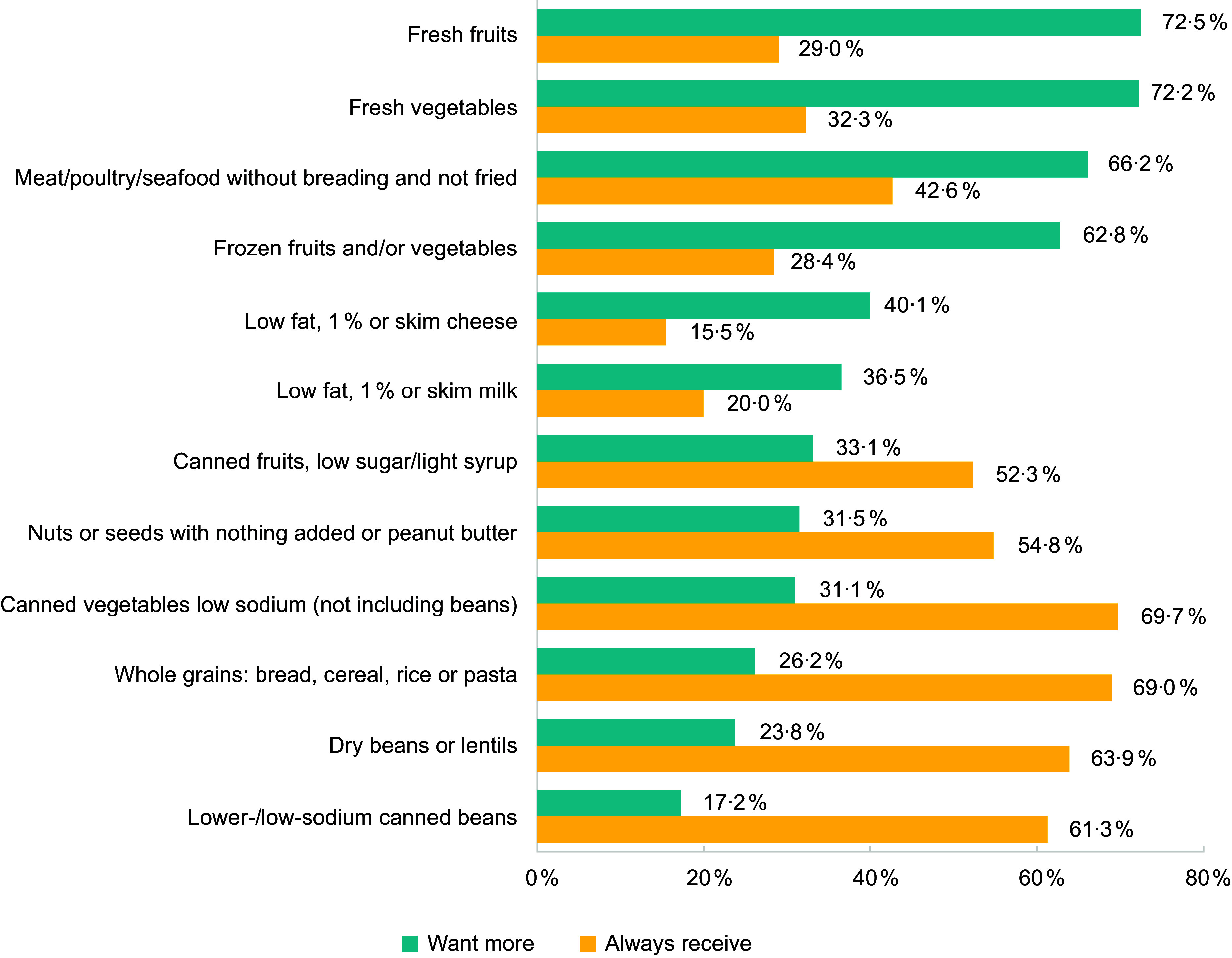
Client food preferences and reports of foods always/almost always received (%), 2022.

Figure 3 shows 2022 survey responses comparing clients’ food preferences and the percentage of respondents reporting that they always/almost always receive those foods from the food pantry. As depicted in the figure, an inverse pattern exists. Most clients wanted fresh fruits, fresh vegetables, frozen fruits and/or vegetables and meat/poultry/seafood without breading and not fried more often. These same foods were among the least likely to be reported by clients as always/almost always received. In contrast, four foods that most clients reported always/almost always receiving were less desired (primarily shelf-stable items including canned vegetables (low-sodium), whole grains, dry beans or lentils and lower-/low-sodium canned beans).

Table 5 shows respondents’ health-related characteristics. In 2021, food insecurity prevalence was 61·3 %. Nearly half of respondents (42·6 %) had a BMI ≥ 30, indicating obesity, and 21·3 % had a BMI the healthy range. In 2021, 34·8 % of households had at least one household member diagnosed with type 2 diabetes and 72·9 % with hypertension. About 31 % of respondents indicated their own health status was poor or fair. There were no significant changes to these data at follow-up.

## Discussion

Funded by CDC’s REACH programme, a local health district and a small, rural food pantry partnered to implement PSE strategies including a nutrition policy, local partnerships for procuring fresh produce and expanded refrigeration capacity at the pantry. Additionally, the partners conducted a community awareness campaign promoting the food pantry and a monthly client nutrition education programme. In interviews, partners discussed the importance of relationship building and trust as key to working together towards mutual aims and overcoming barriers including limited resources and capacity. Consistent with evaluations of PSE change interventions in other rural communities^(35,36)^, partners noted that change takes time in rural communities and REACH staff encouraged incremental change in working with food pantry leaders.

We found that limited organisational capacity was overcome through robust partnerships at multiple levels, consistent with literature on PSE changes in food pantries and frameworks, such as the Interactive Systems Framework, that address capacity across delivery systems^(23,24,37–40)^. Thus, implementation relied on partners working together and co-determining project pace and scope. Food pantry staff had deep ties to the local community and relevant personal experiences that facilitated their work. Finally, REACH staff volunteering in the pantry built relationships and trust among pantry volunteers and leadership, allowing them to address mutual aims and formalise the relationship with a memorandum of understanding solidifying their commitments.

This evaluation’s logic model was designed to produce the short-term outcome of adoption of a nutrition policy. REACH partners articulated how simultaneously implementing system and environmental change strategies increased organisational capacity to implement the policy, consistent with previous work^(41)^. For example, Hancock County is relatively remote, far from a major highway; this geographic isolation presents challenges for food delivery systems, thus making procurement of local produce critical. REACH staff partnered with local farmers and provided funding to ensure that fresh, local produce was provided in the monthly food boxes. During the grant period, the regional food bank began sending more produce as well. The food pantry initially had limited cold storage capacity and would have been unable to properly store increased amounts of produce and other perishables without the food bank’s donation of two large refrigerators. Despite resources provided by REACH, the added expense of purchasing additional produce, including paying local farmers a fair price and sometimes paying retail price in stores, constrained project budgets, at the same time client volume was increasing. This may be a limiting factor for small, volunteer-run food pantries in their ability to provide large amounts of fresh produce to clients.

Another key system-level partner, the food bank could be a key factor in the success of food pantry nutrition initiatives. In late 2023, after the REACH grant ended but perhaps a downstream effect of their work, the regional food bank began implementing the Healthy Eating Research nutrition guidelines^(28)^. These guidelines can support informed decision-making by food bank and food pantry donors, users, staff and volunteers in using common evidence-based metrics to identify nutritious foods. Ultimately, these guidelines can help increase the nutritional quality of foods distributed to clients, increasing nutrition security and reducing income-based gaps in the nutritional status of Americans^(20,28)^. Implementation of these guidelines at the food bank further supports REACH partners’ efforts to increase distribution of healthy foods at the pantry. These are just a few ways that implementing PSE change strategies can reinforce one another, enhancing overall implementation and outcomes.

Similarly, co-implementing individual-level nutrition education strategies amplifies PSE strategy effectiveness and vice versa. A review of mixed-methods nutritional intervention studies provides support for multilevel interventions in increasing access to healthy foods, in accordance with the socio-ecological model^(42)^. Furthermore, nutrition education can help clients make healthy choices outside of the food pantry context^(43)^.

Despite persistent efforts of partners to improve the nutritional quality of foods distributed, client-reported changes in foods always/almost always distributed from 2021 to 2022 were mixed, with some significant changes in expected and client-desired directions. Surprising, though, is the lack of perceived increase in frequency of fresh produce received, given partners’ efforts to provide fresh produce every month and to promote client awareness of those changes. There are a few potential explanations for the lack of perceived changes overall. The nutrition policy largely codified existing efforts to provide healthy foods at the food pantry and included needed exceptions. The modest policy scope might have limited potential for client impacts despite partners’ described efforts. A more ambitious policy, if feasible, might have achieved measurable client-related outcomes. Related, the 1-year client survey follow-up period may have been too brief to capture changes in client perceptions or was perhaps too long for recall. Although we lacked access to objective data on food box contents, that information could increase our understanding about the true extent of change following policy implementation. However, a review of twelve food pantry interventions (not policy-specific) found that studies with modest intervention strategies, short follow-up periods and subjective assessment methods determined that interventions were effective in improving diet-related outcomes, suggesting that such interventions and evaluation methods can still demonstrate change^(16)^. Finally, the food pantry experienced substantial growth in client volume during the study period; yet, as demand increased, budgets did not keep pace. Consequently, the food pantry served more households with the same amount of resources. While more clients may have been theoretically reached by the nutritional policy, the amount or frequency of produce and other healthy foods received might have stayed the same or decreased due to resource constraints.

At both time-points, clients reported high interest in receiving fresh, healthy foods (e.g., fresh/frozen produce and animal proteins). REACH staff and pantry partners shared anecdotal reports that clients appreciated the changes. This finding is consistent with prior research that has demonstrated client demand for healthy foods^(32,44)^. Combined with high desire for healthy foods, this suggests that their efforts were welcome and should continue. Simultaneously, large majorities reported always/almost always receiving shelf-stable goods (e.g., canned vegetables and canned/dried beans), and relatively small proportions reported wanting more of those foods. Understandably, clients might not want more of the types of foods that they already receive monthly, especially those that can be stored for longer periods of time than perishable foods. REACH staff aimed to increase the acceptability of beans and lentils by providing recipes, but these foods might still have been less preferable to meat as a protein source.

This study has several limitations. Although this evaluation was intentionally designed to focus on understanding factors affecting PSE change implementation in a small rural food pantry in a racially minoritised area, these findings may have limited generalisability as a result. However, findings may be especially meaningful to practitioners working in similar communities. The passive survey recruitment methods may have introduced unknown response bias. Lack of objective food box data limits our ability to interpret change in foods clients reported always/almost always receiving. Self-report survey data are subject to several forms of bias, including social desirability bias. In addition, our brief survey limited our ability to ask more detailed questions. This evaluation was designed primarily to assess the nutrition policy. Although we learned of system and environmental changes through our evaluation work, some findings related to system and environmental changes are consequently limited compared to the nutrition policy. This survey did not find any significant changes in health outcomes resulting from changes at the food pantry during the study period. This is likely due to the relatively short study period, limited contribution of food box contents to clients’ overall diets and extent of perceived changes to food box contents during the study period. Finally, this study began in the first year of the COVID-19 pandemic. The 2021 survey in particular may have captured above normal food insecurity and need created by the pandemic. Although we were unable to collect objective food box data, the mixed-methods proved invaluable. The information obtained through implementer interviews helped contextualise the survey findings, particularly the lack of client perceived changes.

Food pantries are an important source of food for individuals and families experiencing food insecurity and should be acknowledged as a key delivery system setting for improving nutritional security among rural communities. Increasing the nutritional quality of foods provided to food pantry clients can contribute to improved health outcomes in the long-term and improved client satisfaction in the short term. As part of the charitable food system, food pantries are chronically under-resourced and have modest capacity. Despite these challenges, partners were able to work together and make changes that if expanded and sustained could support improved client access and diets. To achieve such changes, considerable dedication of resources to strengthen and sustain this important work will be needed.
